# Impact of Directive 2013/11/EU on Consumer ADR Quality: Evidence from France and the UK

**DOI:** 10.1007/s10603-018-9394-z

**Published:** 2018-11-15

**Authors:** A. Biard

**Affiliations:** Erasmus School of Law, Erasmus University Rotterdam, Burgemeester Oudlaan 50, Postbus 1738, 3000 DR Rotterdam, Netherlands

**Keywords:** ADR, ODR, Directive 2013/11/EU, Mediation, Quality, Ombudsman

## Abstract

One of the objectives of Directive 2013/11/EU was to promote high-quality consumer alternative dispute resolution (ADR) schemes in the European Union (EU) through the creation of certification processes and regular monitoring by Member States. To obtain and keep certification, ADR bodies must continuously comply with several binding requirements set down in the Directive testifying–among other things–of their impartiality, expertise, transparency, accessibility, as well as of the fairness, timeliness and effectiveness of their procedures. The objective of this regulatory architecture was to trigger some long-term effects on the procedural design and functioning of ADR bodies and to enhance their credibility and legitimacy vis-à-vis consumers and traders. As such, the new rules have aimed to respond to the criticisms sometimes expressed about the way ADR providers operate, in particular concerns regarding schemes’ lack of independence, limited accountability and possible effects on due process. Yet, doubts have been expressed about the ability of the Directive to secure a consistent approach fully supporting high-quality ADR in the EU. This paper intends to test these doubts against facts and evidence. Based notably on replies to a questionnaire sent to Competent Authorities, it zooms in on experiences in two Member States, namely France and the United Kingdom (UK) (more specifically for the latter in the civil aviation and non-regulated sectors). It highlights how the binding quality criteria have been working in practice and the impacts that the Directive has had on ADR bodies in those Member States and sectors. It sheds light on several persisting issues, and makes some policy recommendations, which may be relevant for policymakers not only in France and the UK, but also in other Member States and at the EU level when further developing a sustainable framework for high-quality ADR. In 2019, the European Commission is expected to publish a report on the implementation of the Consumer ADR Directive in all Member States. This contribution may be viewed as a first small step in that direction.

Directive 2013/11/EU^1^ (hereafter, “Consumer ADR Directive” or “Directive”) has intended to promote high-quality consumer alternative dispute resolution (ADR) schemes in the EU through the creation of approval processes and regular monitoring. To obtain and keep certification, ADR bodies must continuously comply with several binding requirements set down in the Consumer ADR Directive testifying–among other things–of their impartiality, independence, expertise, transparency, accessibility as well as of the fairness, timeliness and effectiveness of their procedures. The Directive builds on and further consolidates the architecture established by two Recommendations of the European Commission in 1998 and 2001 (EU Commission 1998; 2001), which set down minimum quality requirements for out-of-court procedures but had to be revised to consider new developments in ADR sectors (Hodges et al. 2012; Hodges and Creutzfeldt 2013). The underlying objective of this new framework was to trigger some long-term effects on the procedural design and functioning of ADR bodies, and to enhance their credibility and legitimacy vis-à-vis consumers and traders (Cortés et al. 2016). As such, it aimed to respond to the criticisms sometimes expressed about the way ADR providers operate, in particular concerns regarding schemes’ lack of independence, limited accountability and possible effects on due process (Lindblom 2008; Civic Consulting 2009; Eidenmüller and Engel 2014; Wagner 2014).

The Directive has followed a minimum harmonization approach. It has sketched a framework that Member States were free to further complement to ensure a higher level of consumer protection.^2^ The Directive also gave Member States some leeway to create their own certification and monitoring processes reflecting and tailored to the peculiarities of their national ADR landscapes. As a consequence, ADR certification and monitoring tend to diverge significantly across the EU. Some Member States (e.g., Italy, the UK) have appointed several “Competent Authorities” in charge of certifying and monitoring ADR providers, while others (e.g., France, Belgium) have designated one single authority responsible for all schemes regardless of the sector in which they operate. Profiles of those authorities also differ: Some Member States have given this role to Ministries of Economy (e.g., Belgium, Luxembourg, Romania), Ministries of Justice (e.g., Finland) or to sectoral authorities (e.g., Italy, UK). These authorities should ensure that the privatisation of consumer dispute resolution takes place within certain boundaries complying with key justice requirements and preserving the rights and interests of consumers.

Yet, concerns have been expressed about the ability of the Directive to secure a fully coherent and consistent approach supporting high-quality ADR throughout the EU (Biard 2018; Cortés 2015a; Kirkham 2016; Loos 2016). This paper intends to test these concerns against facts and evidence. Based notably on replies to a questionnaire sent to Competent Authorities, it aims to understand how the binding quality criteria have been working in practice, and to highlight some obstacles that Competent Authorities have faced when certifying and monitoring ADR bodies. It seeks to further understand the impact that the new framework has had on ADR schemes, and to shed some light on the way ADR bodies have responded to the quality requirements laid down in the Directive and national legislations. In 2019, the Commission is expected to publish a report on the implementation of the Consumer ADR Directive in all Member States.^3^ This contribution may therefore be viewed as a first small step in that direction.

The rest of this paper is divided as follows: It first reviews the EU framework supporting high-quality ADR (1). It then zooms in on national experiences in two Member States, namely France and the UK, and presents the methodology used for collecting data. Regarding the UK, the paper focuses in particular on the civil aviation and non-regulated sectors, which are sectors that have been through significant changes since the implementation of the Consumer ADR Directive (2). The paper then looks to the future: It highlights some key ongoing challenges (3) and make several policy recommendations that policymakers may further consider when developing a sustainable framework for high-quality ADR (4).

## The EU Framework for High-Quality ADR

Progressively, the EU has sketched a framework and drawn up quality requirements to ensure that ADR bodies comply with key justice requirements (1.1). Yet some doubts tend to persist as regards the capacity of EU initiatives to secure a coherent and consistent framework sustaining high-quality ADR (1.2). It is therefore necessary to test these concerns against facts and evidence and to analyse how those quality requirements have been working in practice (1.3).

### EU Initiatives for High-Quality Consumer ADR

In 1996, the European Parliament called on the European Commission to adopt minimum quality requirements for ADR schemes with the intent to enhance “the impartiality of the body passing judgement, the efficiency of the procedure and the publicizing and transparency of the proceedings” (EU Parliament 1996). This resulted into two Recommendations of the Commission published in 1998 and 2001, and respectively applying to ADR within and outside court systems (EU Commission 1998; 2001). Despite the Recommendations’ added value and influence, a binding legislative instrument turned out to be necessary as a significant number of schemes continued to be “not in line with core principles laid down by the two Recommendations” (EU Commission 2011). Moreover, there was no obligation for national public authorities to regularly monitor the effectiveness of ADR providers (EU Commission 2012). In parallel, contexts and environments in which ADR schemes operated had transmogrified with an ever-growing role played by the Internet and a multiplication of cross-border transactions (Hodges et al. 2012).

In 2013, the Consumer ADR Directive established a revised regulatory framework for consumer ADR with the creation of certification and monitoring processes (Cortés 2015b; Creutzfeldt and Hodges 2014; Page and Bonnyman 2016). Schemes seeking certification must now prove compliance with several binding quality criteria showing, among other things, their independence, impartiality, fairness and expertise.^4^ As some observers have argued, these requirements tend to mimic and to translate into the ADR sphere some of the core principles constituting the right to a Fair Trial, as laid down in Article 6.1 of the European Convention on Human Rights (Poli 2015). Member States were also free to go beyond the minimum criteria of the Directive and could impose additional requirements on ADR entities to ensure higher level of consumer protection. Although the Directive did not make certification compulsory for all ADR providers, certification is expected to come with several advantages (Biard 2018). In particular, it contributes to promote schemes’ visibility since traders can only inform consumers about certified ADR entities when disputes arise, and only certified ADR can be listed on the EU Online Dispute Resolution (ODR) Platform. Certification should also act as a sign of quality (a “trust-mark”) incentivising consumers and traders to refer complaints to approved ADR bodies.

In this context, Member States were required to appoint one or several “Competent Authorities” standing at the core of the new framework.^5^ The initial proposal for a Directive published by the EU Commission did not allow for more than one authority per Member State. This possibility was introduced later on and justified on the ground that “in order to reflect different sectoral or geographical approaches to ADR, it is necessary to allow Member States to designate more than one Competent Authority” (EU Parliament 2012). Today, two main approaches have been followed by Member States: a horizontal (or centralized) one where one single authority is in charge of certifying and supervising all ADR entities across all sectors, and a vertical (or decentralized) one where certification and monitoring are performed by regulators, depending on the sector in which schemes operate (Biard 2018). In the latter model, one authority is also assigned the role of coordinating the Competent Authorities group and serves as the contact point for the European Commission.

National Competent Authorities perform a wide range of administrative and policy duties.^6^ They review certification applications submitted by ADR entities. They assess the quality of ADR providers from the point of view of justice and their performance in providing quick, accessible and low-cost dispute resolution mechanisms for consumers and traders. They conduct regular audit exercises, collect data and factual information about ADR providers, and disseminate this information to the European Commission and the wider public. They draw up a list of all certified ADR bodies and must keep the list updated. They can withdraw certification to schemes that fail to comply with the quality criteria on an ongoing basis. Finally, they contribute to policy-making by publishing regular reports highlighting best practices and supporting policy recommendations. All in all, Competent Authorities thus operate as regulators of ADR sectors (Biard 2018).

### Persisting Doubts

The minimum harmonization approach followed by the Directive has triggered some questions and concerns about its capacity to fully secure a consistent framework supporting high-quality consumer ADR in the EU (Cortés 2015a; Kirkham 2016; Loos 2016; Biard 2018). Some of these concerns can be summarized as follows:

- *Vagueness of the quality requirements as listed under the Directive*. The Consumer ADR Directive is a framework Directive. The way the quality requirements have been described had to be broad enough to cover any forms of ADR schemes operating in any sector. While permitting some flexibility in their implementation, the quality requirements are also likely to remain a low common denominator not fully meeting consumers’ expectations. For example, the Gambling Commission in the UK highlighted in 2017 that “one year on, it has become apparent that the requirements of the regulations were written broadly to meet the needs of a range of different sectors. As a result, they do not fully cover all consumer expectations in the gambling sectors” (Gambling Commission 2017, p. 15). As the Commission further added, “the ADR regulations require providers to meet basic standards around transparency, clarity, independence, etc. However, those requirements may not be sufficient to provide service levels that meet customer needs and need to be augmented with a framework of guidance, standards and measures to improve standards in the gambling sector” (Gambling Commission 2017, p. 20).
- *Behaviour and degree of scrutiny of Competent Authorities*. Another issue regards the behaviour of Competent Authorities during the review of certification applications and subsequent quality checks. For cost reasons or limited capacity, or simply because the authority might not fully be familiar with the way ADR schemes operate, some authorities might be tempted to briefly review the information submitted by applicants and might merely “tick the box” when reviewing the quality criteria. Conversely, others might be eager to conduct in-depth assessment and might carefully scrutinize the functioning and procedural design of applicants. Differences in behaviour and in degrees of scrutiny between Competent Authorities mean that certified ADR providers with uneven quality can coexist across the EU. Furthermore, the Directive has required Member States to regularly monitor certified ADR providers. Experience has shown that this was not always the case in the past. For example, in 2009, a study on ADR reported that it was “clear that notifying authorities monitor[ed] compliance of ADR schemes with the Recommendations often only at the time of notification. A regular follow-up monitoring appear[ed] to be the exception, and was only reported from Belgium” (Civic Consulting 2009, p. 123). Obviously, this observation was made in the context of the non-binding Commission recommendations. However, it remains to be seen whether the behaviour of Competent Authorities has changed as a result of the Directive.
- *Diversity of quality criteria and possible risks of regulatory shopping*. A third issue is linked to the diversity of quality standards across the EU and to the fact that traders established in one Member State may be covered by a scheme established in another Member State.^7^ This situation is likely to create a risk of regulatory ADR shopping in which traders signpost their consumers to cheaper ADR bodies that are certified in other Member States, but are also applying lower requirements than the domestic ones. This may lead to a race to the bottom jeopardizing the efforts of those Competent Authorities that are imposing higher quality criteria on their ADR providers as an attempt to step up consumers’ protection.
- *Structure of national monitoring architectures and possible lack of consistency between certification and monitoring practices*. The structure of monitoring architectures–in particular the vertical model in which several Competent Authorities within a same country operate jointly and independently–has been criticized. An author, for example, argued that “in the multiple Competent Authority model adopted in the UK, no one body has the data to collate best practice on ADR and there is only a minimal incentive for Competent Authorities to compile such information or act as a force for higher standards” (Kirkham 2016, p. 320). The question is whether–and if so, how–Member States that are relying on such vertical approaches have managed to ensure homogeneity and consistency between different Competent Authorities’ practices.
- *Multiplication of ADR schemes and survival of non-certified schemes*: In some Member States, ADR has become a market with new opportunities for private entrepreneurs. Sometimes, this may have contributed to a fragmentation of ADR sectors, ultimately jeopardizing consumers’ visibility and creating confusion. Moreover, as pointed out earlier, the Directive did not make certification compulsory for all ADR entities. Therefore, as an author noted, “it is also possible that some ADR providers that comply with the legal requirements decide not to apply for certification and enjoy more regulatory freedom instead” (Miquel 2016, p. 179). In some Member States at least, this means that non-certified ADR entities can continue to operate alongside certified ones, thus creating two-tier ADR landscapes.

Since ADR models and landscapes vary significantly across the EU, the risks presented above might not be equally present in all Member States. However, taking them into consideration appears to be a necessary step for designing a sustainable framework for high-quality ADR in the EU.

### Testing the Doubts: Methodology and Limits

This paper intends to review some of the problems listed above against facts and evidence, and analyses how certification and monitoring processes have been working in practice. Collection of data was carried out through different sources of information. Between January and May 2018, a short online questionnaire entitled *Monitoring Consumer ADR Quality and the Role of National Competent Authorities: The Way Forward* was sent to 29 Competent Authorities in 17 different Member States.^8^ Targeted countries covered different geographical regions of the EU and were likely to vary significantly in their degree of familiarity with Consumer ADR.^9^ The survey included open and multiple-choice questions (18 questions in total) investigating three main topics:

1. Competent Authorities’ experience with certification and monitoring techniques: This category included questions about the type of information requested from ADR schemes when seeking certification, whether authorities impose additional quality requirements on top of those listed in their legislations, potential difficulties met during the review of certification applications, periodicity of control checks, and whether checks are performed by surprised and/or in collaboration with schemes.
2. Relationships between Competent Authorities and other stakeholders: This category investigated the ecosystem in which Authorities evolve, in particular whether they have contacts and exchanges (and if so, with which frequency and on which topics) with other national or EU Competent Authorities, sectoral regulators, and judges or the judiciary.
3. Competent Authorities’ views on ongoing developments in their respective ADR sectors, their opinions on the roles of Competent Authorities and tools likely to enhance ADR quality: This category included questions about their views on the role of Competent Authorities for further organizing and structuring ADR sectors, and questions on possible tools for facilitating their interventions in the future.

Designing a questionnaire likely to trigger replies from stakeholders is a challenging task given the small amount of time that respondents usually accept to devote to this type of exercise. For this reason, the survey had to be concise, and could therefore not explore all facets of Competent Authorities’ daily work. It is important to clarify here that the questionnaire did not per se intend to collect all information about Competent Authorities’ practices and experience but rather to trigger further follow-up in-person interactions and discussions. At the end of the survey, respondents were therefore questioned about their availability to further explain (or nuance) their replies by telephone, e-mail or during a meeting. The objective of these follow-up discussions was to better understand the policy and regulatory backgrounds in which respondents operate and to replace their responses in broader contexts.

Many Competent Authorities did not reply to our invitation to complete the survey. Some did acknowledge receipt but ultimately did not fill in the questionnaire. Some did reply but then did not take part in follow-up discussions, or provided limited additional information afterwards, making the use and interpretation of their responses ultimately difficult without a clear understanding of broader policy and regulatory contexts at national level. This paper focuses on the results collected with three Competent Authorities, one operating in France (*Commission d’Evaluation et de Contrôle de la Médiation de la Consommation*) and two in the UK (Civil Aviation Authority and Chartered Trading Standards Institute).^10^ These Authorities agreed to complete the survey and provided meaningful contextual and policy information. As a follow-up, in-person meetings and requests for clarifications based on their replies to the questionnaire took place with the Directorate General for Competition, Consumer Affairs and Fraud Prevention (*Direction Générale de la Concurrence, de la Consommation et de la Répression des Fraudes* (DGCCRF)) in France (one meeting and exchanges by e-mail), the Civil Aviation Authority (one meeting and exchanges by e-mail) and the Chartered Trading Standards Institute (exchanges by email). To complete the analysis, policy documents published by these Authorities as well as reports from certified ADR schemes in these two countries were considered so as to further understand the impact that certification and monitoring has had on schemes’ structures and functioning and to better understand the nature of their relationships with Competent Authorities. A specific attention was given to reports published between 2015 and 2017 as most of ADR schemes sought certification within this period of time.

This study also has several limitations, which should be clarified upfront. First, data collected about ADR certification and monitoring that are hereafter presented only concern France and the UK. We acknowledge that, although some similarities may well exist in other Member States, results collected can obviously not be extrapolated to all Member States as national situations are likely to vary in the EU for domestic reasons. However, it is believed that some lessons learnt from these two Member States might potentially also be of interest for other Member States and instructive for policymakers. Second, as regards the UK, we acknowledge that data do not depict the situation of ADR monitoring and certification across all economic sectors in this country but only in the civil aviation sector and in non-regulated sectors. As explained below, there are sectors where ADR is more institutionalised (for example, in the financial sector).^11^ This being said, civil aviation and non-regulated sectors remain interesting objects of research because limited research has been conducted on the development of ADR in these fields after the implementation of the ADR Consumer Directive (see, however, Creutzfeldt and Berlin 2016 for the aviation sector). These sectors have also been (and are still going) through significant changes since the implementation of the Directive. Third, investigating the work of Competent Authorities and the issue of ADR quality is like shooting at a moving target. Practices and techniques of Competent Authorities are dependent on national policy contexts and ADR landscapes, which themselves are changing and evolving constantly. As hereafter highlighted, Competent Authorities are often adapting their behaviour to new regulatory frameworks. For this reason, data presented in this paper capture certification and monitoring by Competent Authorities as it has been performed between the entry in force of the Consumer ADR Directive and 2018.

A final remark concerns the presentation of the data throughout the paper. Responses from the three Competent Authorities have been put together in Tables 2, 3, 4, 5, 6, 7, 8, 9, 10, 11 and 12. This comparative approach is useful to highlight existing convergences and divergences between Competent Authorities’ practices.

## Developing High-Quality ADR at Member States Level

The paper zooms in on experiences in France (2.1) and the UK (2.2) as an attempt to further understand the impact of the Directive at Member States’ levels.

### France

#### Background

The Consumer ADR Directive was transposed in France by Ordinance 2015-1033 of 20 August 2015, and further specified by two implementing decrees (decree 2015-1382 of 30 October 2015 and decree 2015-1607 of 7 December 2015). All provisions are consolidated in the French Consumer Code (*Code de la consommation*).^12^ France already had experience with ADR in several economic sectors (such as banking or transports) long before the implementation of the ADR Directive (Bernheim-Desvaux 2016; Creutzfeldt 2016; Mallet-Bricoud 2015). The new rules, which came into force on 1 January 2016, have generalized consumer ADR to all sectors. When certified, schemes are referred to and listed as *Médiateur de la consommation*.^13^ They must provide free of charge services for consumers.^14^ All traders (regardless of their size) operating in all economic sectors must signpost consumers towards an ADR provider of their choice.^15^ Traders are however not bound by the outcome of the ADR process.

The French ADR landscape contains ADR entities operating on a number of different models (Cadiet and Clay 2017). It is composed of two public ombudsmen (*Médiateur de l’Autorité des Marchés Financiers* and *Médiateur National de l’Énergie*) established by statutes, sectoral entities (e.g., insurance ombudsman), bodies linked to professional organizations and various entities of different types including associations of mediators and other private schemes.^16^ One peculiarity of the French ADR sector regards the high number (approximately 30) of in-house mediators (*médiateur d’entreprise*), which are schemes created in the 1990s, and embedded into large companies or banks (e.g., SNCF (Société nationale des chemins de fer français - the French national railways company), EDF (Electricité de France), Engie, La Poste, BNP Paribas etc.). Initially, the proposal for a consumer ADR Directive did not include in-house mediation, and the European Commission was reluctant to recognize this peculiar type of ADR. After strong lobbying, France however successfully managed to have them included in the scope of the Directive. This does not necessarily mean that the French government is content with the long-term existence of in-house mediators. As a consequence of the Directive, in-house mediators are nonetheless now subject to stricter requirements strengthening their autonomy and independence.^17^ Such requirements are likely to make the creation of in-house mediators more difficult in the future (Grison 2018). Finally, some networks have been created to facilitate the exchange of best practices between ADR bodies. In particular, the *Club des médiateurs des services au public*, created in 2002, gathers approximately 25 schemes and includes public ombudsmen, in-house mediation services and other entities.

#### Certification Process and Impact on ADR Schemes

ADR bodies are certified, and afterwards monitored, by a new public entity especially established for that purpose. It is known as the Evaluation and Monitoring Commission for Consumer Mediation (*Commission d’Évaluation et de Contrôle de la Médiation de la Consommation* (CECMC)).^18^ The CECMC is composed of 18 members appointed by the order (*arrêté*) of the Ministry of Economy.^19^ It includes consumers’ and traders’ representatives, qualified personalities (e.g., academics) and some high-profile judges. The Commission is presided by a former judge of the criminal Chamber of the Court of Cassation. The rather unique profile and composition of the French Competent Authority may be explained by domestic reasons, in particular the desire to give to the CECMC a high moral authority to enhance consumers’ confidence and to strengthen its independence, in particular vis-à-vis in-house mediators historically connected to large corporations. The Directorate General for Competition, Consumer Affairs and Fraud Prevention (*Direction Générale de la Concurrence, de la Consommation et de la Répression des Fraudes* (DGCCRF)), attached to the Ministry of Economy, serves as its secretariat (Bernheim-Desvaux 2017).

Obtaining certification is free of charge in France. The CECMC and DGCCRF provide guidance on their websites for applicants.^20^ In practice, DGCCRF dedicated team (approximately five people) receive, process and issue opinions on the applications that they receive. The application is then submitted to the CECMC for decision. Applications are reviewed through the information submitted by the applicant, oral hearings and, when applicable, onsite visits (Table 2). Between 2015 and January 2018, the CECMC reviewed 95 applications, of which 70 were certified.^21^ In July 2018, the number of certified ADR schemes reached 80, covering approximately 86% of all economic sectors.^22^

Certification has impacted on the functioning of ADR bodies in many ways. Several schemes have improved their transparency, internal capacity, and the quality of the information provided to consumers. The Financial Markets Authority Ombudsman (“AMF Ombudsman”), which was the first entity to be certified in 2016, has revised the online materials available to consumers, and added information and tools for users (Médiateur AMF 2016), which in particular now include an online referral form to contact the Ombudsman more easily. Several schemes have amended their dispute resolution schemes. For example, the public energy Ombudsman (*Médiateur Nationale de l’Énergie*) has put an end to its “second-chance” rule, which allowed complaints that had not been at least superficially processed by the trader to be sent back to the trader for additional examination. This rule was used to preserve the Ombudsman’s resources and to maintain greater scrutiny for more complex cases. As the Ombudsman’s managing director explained, the Ombudsman has “complied with the Commission’s requirement to process all referral procedures in depth” (Médiateur de l’Énergie 2016, p. 11). Several schemes also have increased their staff to facilitate the resolution of disputes within 90 days, as requested by the ADR Directive (see, for instance, Médiateur de l’assurance 2016). For example, the average processing time of complaints by the telecom Ombudsman (*Médiateur des communications électroniques*) has decreased from 135 days in 2016 to 80 days in 2017 (Médiateur des télécommunications électroniques 2017). In-house mediators have also reviewed their procedures. For example, *Médiateur du Groupe La Poste* has amended its Charter and revised its website, which now includes a mechanism allowing users to upload documents online so as to facilitate the treatment of their claims (Médiateur du Groupe La Poste 2016).

#### Challenges

The new certification process has turned out to be more complex, demanding and time consuming than initially expected. Key problems faced by the CECMC and DGCCRF when reviewing applications are presented in Table 3. The CECMC does not impose additional quality requirements that those listed in the ADR Directive and implementing legislations on schemes seeking certification (Table 4). From the very beginning, the CECMC had to deal with several challenges pertaining to the complexity of ADR sectors in France and to the wide diversity of existing schemes. The CECMC also had to ensure full coverage in a short period of time so as to ensure that certified ADR entities could be available in all economic sectors. This was made difficult and particularly challenging in the absence of a residual ADR entity covering gaps in ADR coverage.

In parallel, DGCCRF services had to provide a significant amount of assistance to applicants. For instance, they helped applicants when drafting (or re-drafting) their charters and adapting their websites to new legal requirements. The CECMC also drafted–and made available on its website–a template collaboration agreement to regulate relationships between ADR schemes and traders.^23^ It must be noted that the CECMC has high expectations, in particular as regards the independence of ADR schemes. Several applications were rejected because the applicants failed to meet the requirements. The CECMC also carefully analyses how ADR entities meet the quality criteria *in concreto.* It scrutinizes applicants’ business plans and verify the economic viability of their activities, their charters, the contents and nature of agreements signed with traders, their level of legal and technical expertise, and the absence of conflicts of interests. Applications from several in-house mediators have required several back and forth between applicants and the CECMC. According to data communicated by the CECMC in July 2018, 66% of received applications have required one meeting before the CECMC, 23.6% required two meetings and 10.4% up to three meetings.

#### Monitoring

The CECMC and DGCCRF perform monitoring exercises and control checks once a year in cooperation with certified schemes (Table 5). After two years mainly dedicated to certification, the CECMC and DGCCRF may progressively strengthen and multiply their monitoring activities. They also conduct additional controls or can request clarifications from certified entities when they identify problems or when consumers highlight issues relating to the quality of services offered by a scheme. For example, consumers have approached the DGCCRF to complain about the alleged lack of independence of certain entities or to complain about delays in complaint-handling processes. In some circumstances, the CECMC and DGCCRF contacted ADR bodies and requested additional clarifications about practices. In some situations, the CECMC also heard schemes during plenary meetings. In one case, the CECMC took temporary measures against a non-compliant scheme.^24^ CECMC’s oversight sometimes has triggered heated discussions between the Competent Authority and some ADR schemes. The CECMC seems however willing to continue in this way so as to ensure high-quality ADR bodies and to enhance confidence of consumers.

#### Relationships Between the Competent Authority and Stakeholders

As shown in Table 6, the CECMC has regular contacts (i.e., more than two times per year) with sectoral regulators. Occasionally, it also has exchanges with judges or the judiciary, in particular for clarifying legal issues. Such connections with the judiciary (a situation that is peculiar when considering the absence of contacts between the judiciary and the two other Competent Authorities surveyed below in the UK) might be explained by the original composition of the CECMC, which is itself composed of several judges. The CECMC also has occasional contacts with Competent Authorities from other Member States (one to two times per year). Certified ADR schemes regularly (more than two times per year) assist the Competent Authority in identifying systemic issues or sectoral problems. In particular, schemes highlight difficulties faced in their respective sectors, and refer identified problems to the CECMC.

#### Ongoing Developments in ADR Sectors

The questionnaire intended to further understand the views of Competent Authorities on the development of ADR sectors, and investigated whether they regarded the existence of multiple schemes as an added value or, on the contrary, as an inconvenient for sectors (Table 7). Response from the CECMC was nuanced. One the one hand, the presence of multiple schemes in a sector may foster competition. On the other hand, the CECMC also highlighted the added value of having one sectoral scheme. One of the advantages of the latter lies in the fact that sectoral schemes have to accept all complaints arising out from their sector (including in situations where no prior agreement has been concluded between the trader and the scheme).

With already 80 certified schemes, consumer ADR in France is a market that remains competitive and highly fragmented (Bernheim-Desvaux 2017). As some consumer organizations have pointed out, the proliferation of ADR entities tends to impair consumers’ visibility and creates confusion (UFC Que Choisir 2016). When the Consumer ADR Directive was implemented, several suggestions were made to introduce some forms of prioritisation and coherence into the French ADR landscape. Recommendations from the Steering Committee in charge of implementing the consumer ADR Directive for example suggested to introduce a hierarchy between ADR providers. As the President of the Committee highlighted, “we tried to ensure the prominence of sectoral and public ombudsmen.”^25^ A report for the French National Assembly in September 2014 also suggested the possibility to deny certification to in-house mediators when there was already a sectoral entity covering the activities of the trader (Assemblée Nationale 2014). The idea did not materialize. Furthermore, in its draft version, the implementing ordinance provided that a trader had to favour a sectoral ADR scheme when the remit of the scheme covered the activities of the trader. However, these initiatives met with resistance from business representatives defending the role and place of in-house mediators. The final version of the ordinance was amended, and now simply provides that traders must always allow consumers to refer their claims to a sectoral entity, when one is available.^26^

This being said, interestingly, in the French energy sector, some forms of hierarchy and cooperation between the public Ombudsman and in-house mediators have been introduced successfully. Experience tends to show that this structure is beneficial for consumers. Under French law, one dispute can normally result in only one mediation process before one ADR body.^27^ There is however one exception to this rule in the energy sector where the public energy Ombudsman (*Médiateur National de l’Énergie*) operates alongside two in-house mediators (*Médiateur d’ENGIE* and *Médiateur d’EDF*). Consumers who are not satisfied with the way an in-house energy mediator has handled their complaint can afterwards still bring their claim to the public energy Ombudsman.^28^ The reverse is however not possible, meaning that consumers cannot bring their claims to an in-house mediator when the claim has first been reviewed by the public energy Ombudsman. In 2015, the public energy Ombudsman signed two agreements with the in-house mediators to further organize their cooperation. As the managing director of the public energy Ombudsman pointed out, “we do not see ourselves as competitors but rather as complementary partners” (Médiateur de l’Énergie 2016, p. 14). Under the agreements, the public Ombudsman and in-house mediators have agreed to transfer cases that are erroneously registered with them because they do not fall under their respective remit (Médiateur National de l’Énergie/Groupe EDF 2015; Médiateur National de l’Énergie/Groupe ENGIE 2015). In terms of outcome for consumers, 13 consumers who had submitted their complaints to the ENGIE in-house mediator sought further assistance with the public energy Ombudsman. The public Ombudsman confirmed the solution proposed by the in-house mediator in seven cases out of 13. In parallel, 84 complaints initially submitted to the EDF in-house mediator were afterwards sent to the public Ombudsman. The public Ombudsman and the EDF mediator shared the same analysis about the dispute in 53% of cases. However, interestingly, in 68% of cases, the two entities did not reach the same conclusions as regards compensation amounts. On average, amounts recommended by the public Ombudsman were four times higher than those suggested by the EDF mediator. As the public Ombudsman’s managing director ultimately highlighted, “even if companies do not fully follow our recommendations, most consumers obtain more than with the solutions initially proposed” (Médiateur de l’Énergie 2016).

By contrast, the architecture of the ADR landscape in the financial and banking sectors remains particularly complex and difficult to navigate for consumers. The AMF Ombudsman is the public ombudsman established by law. It has a monopoly in the investment sector but is not competent in the banking and insurance sectors. In practice, this means that the AMF ombudsman can deal with disputes relating to investment services providers, financial investment advisors or listed companies. The AMF Ombudsman also has concluded agreements with some in-house mediators to share its competences, enabling investors to ultimately refer their claims either to the AMF ombudsman or to in-house mediators. Like in the energy sector, a draft version of the ordinance implementing the Consumer ADR Directive included a provision authorizing consumers to refer their claims to the AMF Ombudsman when they were not satisfied about the way private in-house mediators had handled their requests previously. However, after strong criticisms, this provision was ultimately removed from the final text. Investors must now choose to refer their claims either to the AMF Ombudsman or to the other private ADR entities. Strangely enough, rules are thus different before the two public ombudsmen operating in France.

In addition, the AMF ombudsman is not competent in the areas of life insurance, taxation or bank transactions. More than 25 bank mediators and other private entities (sometimes operating at very local levels) cover disputes relating to banking. This scattered landscape is a consequence of a legislation passed in 2001 (Act no 2001-1168 of 11 December 2001 *portant mesures urgentes de réformes à caractère économique et financier* (MURCEF)), which made it compulsory for all credit institutions to appoint a mediator to deal with complaints. Yet the articulation between the AMF Ombudsman and other banking ADR schemes remains far from obvious for consumers. As the AMF Ombudsman highlighted in its 2017 annual report, there is a persisting lack of comprehension among users concerning the scope of competence of the AMF Ombudsman. In 2017, nearly 49% of the requests received by the Ombudsman had to be redirected because they dealt with bank-related requests (e.g., credits, interest rates or life insurance policies) (Médiateur AMF 2017, p. 8). Back in 2014, the AMF Ombudsman already encouraged the creation of a single entity covering disputes relating to banking, insurance and financial sectors (Médiateur AMF 2014, p. 1). The matter was discussed by the inter-ministerial working group on consumer affairs (*Groupe interministériel de la consommation*) but discussions have remained inconclusive to date.

### UK

#### General Background

The Consumer ADR Directive was transposed in the UK by the alternative dispute resolution for Consumer Dispute (Competent Authorities and Information) Regulations 2015 and the (Amendment) Regulations 2015. In a call for evidence published in 2011 by the Department for Business, Energy and Industrial Strategy (BEIS, formerly BIS), the Government sought views on possible suitable candidates to act as Competent Authority (BIS 2011). Several bodies were identified, including the former Administrative Justice and Tribunal Council and the Office of Fair Trading (OFT). However, these two entities were expected to stop their activities before the actual implementation of the consumer ADR Directive. It was finally decided that a system of several Competent Authorities was the best option for the UK (BIS 2014a). The Government indeed took the view that “it would be preferable for the UK to have more than one Competent Authority, because of the number of sector specific regulators who already oversee regulated sectors with an ADR scheme” (BIS 2014b, p. 19). It considered that “unpicking existing statutory relationships between regulators and ADR schemes would not be helpful and requiring ADR bodies to provide similar information to a regulator and a separate ADR Competent Authority would be duplicative and an unnecessary and costly burden” (BIS 2014b).

Like Italy, the UK has adopted a vertical approach to ADR certification and monitoring with several Competent Authorities acting independently. The Financial Conduct Authority (FCA), the Civil Aviation Authority (CAA), the Gambling Commission, the Legal Services Board, the Powys County Council,^29^ the Office of Communication (OFCOM), the Office of Gaz and Electricity Markets (OFGEM) and the Chartered Trading Standards Institute (CTSI), acting on behalf of the Secretary of State, certify and monitor ADR schemes in their respective sectors (CTSI is in charge of non-regulated sectors). In practice, this means that an ADR entity that is approved by one Competent Authority in one particular sector cannot rely upon this approval to operate in another sector. The scheme must each time and successively apply to the right Competent Authority. Several ADR schemes providing services for a range of industries therefore had to go through parallel approval processes with different Competent Authorities. Moreover, depending on sectors, the quality criteria set down in the Directive and implementing regulations sometimes have been complemented with additional sector-specific quality requirements. As shown hereafter, this is for instance the case in the civil aviation sector.

It should be noted that the ADR landscape is still patchy in the UK (Cortés 2018; Gill et al. 2017). Some areas have well-established ADR schemes and are more developed than others. In some regulated sectors, there is only one statutory ombudsman (for example, the Financial Ombudsman Service for the financial sector), and traders are obliged to sign up to its services. In others (e.g., telecom), traders are also obliged to adhere but can choose between several private entities. By contrast, in other sectors, adherence from traders to an ADR scheme remains voluntary (e.g., in the civil aviation sector), and several ADR bodies are competing to attract complaints. Initially, the Government intended to set up a residual ADR scheme to cover disputes not already covered by an ADR provider. In Autumn 2014, it published an invitation to bid to provide these residual ADR services.^30^ However, the call was finally withdrawn because several schemes offered to fill the gaps existing in ADR coverage (BIS 2014b; Cortés 2015a). The Government also decided to set up a helpdesk in collaboration with Citizen Advice to assist consumers when navigating the ADR landscape.

Below, specific consideration is given to ADR in the civil aviation sector, which is a sector that has been through significant developments since the implementation of the Consumer ADR Directive. In non-regulated sectors, ADR is also a work-in-progress in which CTSI acts as the Competent Authority. It also performs the peculiar role of coordinating the Competent Authorities group in the UK.

#### ADR in the Civil Aviation Sector

##### Background

Historically, passengers’ complaints in the aviation sector were handed down by the Airport Transport Users Council (AUC). They were taken over by the Passenger Advice and Complaints Team (PACT) of the Civil Aviation Authority (CAA) in the 2010s. Until recently, the CAA acted as both enforcer for consumer protection rules in the aviation sector and as main dispute resolution body for consumers. However, the increasing volume of complaints and the perceived confusion in the public eyes between the role of consumer enforcement and dispute resolution prompted a reorganization of its complaint handling process. The implementation of the Consumer ADR directive in 2015 provided the CAA with an opportunity to establish a new framework for consumer complaints where “the future of consumer complaints handling in aviation lies not in the Civil Aviation Authority handling individuals’ complaints, but in this important work being done by private alternative dispute resolution (ADR) schemes, such as consumer ombudsmen” (CAA 2016a, p. 2). It should be noted that, compared with initial situation in which the CAA could only make non-binding recommendations with respect to individual complaints, one important evolution introduced by the CAA has been that ADR providers have now the power to make binding decisions. The CAA acts as the Competent Authority in charge of certifying and monitoring ADR schemes handling disputes concerning contractual obligations arising from consumer contracts for air transport services provided to or from UK civil airports. In practice, this covers complaints about airlines, whether or not they based in the UK (CAA 2016a, p. 9).

##### Certification Process and Impacts on the ADR Sector

The CAA has published several guidance documents for applicants seeking certification (CAA 2016b). In April 2017, certification fees had reached £5684 (approximately €6500). Once approved, the entity also must pay an annual oversight fees as a continuation of its approval, which amounts to £13 642 annually (approximately €15 500).^31^ These amounts were calculated on a cost-recovery only basis. The CAA reviews application based on the information submitted by the applicant, feedbacks from consumers and/or consumer organizations and phones discussions (Table 2). Occasionally, the CAA may conduct hearings with applicants and/or onsite visits. The CAA reviews the minimum quality requirements set down in the Consumer ADR Directive and national implementing regulations and also applies more stringent approval criteria for the purposes of ensuring a high level of consumer protection (Table 4). For example, the CAA requires that fees should be kept to a minimum and only be charged where consumers claims are unsuccessful. In any case, fees should not be charged to consumers when complaints relate to access or equality issues. ADR must also be available for the most common types of disputes between passengers and airlines companies, and where the ADR entity is unable to reach a mutually acceptable settlement between parties, they must make a decision that is binding on the airline company (provided that the consumer agrees with the decision).

In May 2018, two bodies were on the list of CAA-approved ADR entities, namely CEDR (approved in January 2016) and Consumer Dispute Resolution Ltd., also known as Aviation ADR (approved in May 2016). Other applicants enquired about certification but withdrew their applications when they realised the extent of the requirements and the practical work involved in the aviation sector. Noteworthy, two other entities (Ombudsman Services and Net Neutrals) also obtained approvals from the CAA respectively in September 2015 and July 2017 but subsequently decided to withdraw from the ADR aviation marketplace because they perceived the sector as not being economically sustainable at that time.^32^ Development of ADR in the aviation sector has been quick. TUI was the first airline to sign up in January 2016, and approximately 70% of the market was covered within a year of the UK ADR Regulations coming into force. In early 2018, 35 airlines and seven of England’s largest airports had signed up to ADR and almost 80% of air passengers journeys were covered by ADR. In the first year, 10 000 complaints were resolved through ADR, 75% of which in consumers’ favour (CAA 2017a). Noteworthy, as industry participation in ADR remains voluntary in this sector, the CAA had also to come up with some incentives for airlines to join an ADR scheme. It notably did so by introducing a new “user pays” charging model for PACT in June 2016. Under the new rules, airlines that have not signed up to an ADR scheme must cover the costs to the complaints made by their passengers to the CAA. By contrast, those airlines that have signed up to an ADR entity are exempted from contributing towards the costs of PACT. Interestingly, there was a notable increase in the number of airlines signing-up from June 2016, at the same time as the CAA’s new complaint handling charge was introduced.

One of CAA’s key priorities has been to encourage full ADR coverage in the aviation sector. It has publicly criticized several airlines (e.g., Jet2, Emirates or Aer Lingus) that remain reluctant to adhere to an ADR scheme because they consider that ADR is “still new and is finding its feet” (Jet2/CAA 2018). The CAA is currently reviewing its position as to whether participation in ADR should be mandatory for airlines. In particular, it has highlighted that “although we will continue our efforts to promote full participation in voluntary ADR, we will also work closely with Government on its aviation strategy and the issue of voluntary versus mandatory participation in ADR” (CAA 2017b, p. 18).

##### Challenges

At a broader level, reviewing the quality criteria set down in the Consumer ADR Directive was perceived as rather a straightforward exercise (Table 3). However, the complexity has lied in the additional quality criteria that the CAA imposes on applicants. Reviewing those criteria has required a lot of time, with needs to go into ADR schemes’ rules. The CAA had to assist applicants a lot. In its 2017 annual report, the CAA also reported that “each of these [certified entities] underwent a rigorous approval process designed by the CAA to ensure that consumers will be provided by an expert, independent and effective mechanism for resolving their complaints. Through the approval process, schemes rules are specified and checked against; funding arrangements examined; impartiality provisions required; financial position considered; and minimum scope assured” (CAA 2017b, p. 15).

One of the CAA’s concerns regarded the necessity to ensure that the higher certification requirements were not ultimately bypassed by traders seeking services from ADR schemes certified in other Member States. Airlines that carry passengers in and out of the UK can indeed signpost consumers to either a CAA-approved entity, but alternatively, can also use any other EU listed ADR body. Yet those EU listed body may not be subject to the same requirements as those imposed by the CAA. As the CAA has highlighted:

“We felt that it was necessary to provide ADR entities with the ability to enforce their decisions on the airline and the requirement for ‘one way’ binding decisions became a central feature of our ADR policy as a result. In contrast, many European Member States had not gone this far in implementing ADR in their own countries, and the decisions of their approved ADR providers were either not binding or binding only if both sides agreed. We were therefore concerned that if airlines were allowed to signpost their consumers to any ADR provider throughout Europe, they might select the least onerous option (so called ‘regulatory option’).” (CAA 2017b)

The CAA has published a policy document in which it explains that it keeps oversight on non-UK ADR schemes and airlines signposting them. Where the CAA considers that the EU listed body does not meet the requirements and failed to ensure (on an ongoing basis) a high level of protection for consumer, it may inform the airline and the relevant national Competent Authority of its concerns (CAA 2016c). Ultimately, the CAA may remove the approval for the airline to signpost consumers to the EU-listed ADR provider.

##### Monitoring

The CAA carries out formal “continuation of approval” processes every year, as well as ongoing oversight, which includes quarterly meetings, frequent data returns and regular checks on the financial stability of the schemes (Table 5). Control checks are conducted either by surprise or in collaboration with the scheme. If the CAA observe non-compliance from an approved ADR entity, it may take several measures including, in ascending order of severity, providing informal advice, publishing guidance, issuing warning letters, requesting information, securing formal commitments or, as a last response, withdrawing the approval (CAA 2016d).

##### Relationships Between the Competent Authority and Stakeholders

Being a part of the CAA, which is the sectoral regulator for civil aviation, CAA’s ADR team has regular contacts with the regulator. It has however no contact with judges or the judiciary. Occasionally, it has contacts and share experience with other Competent Authorities in the UK on various matters to enhance good practice. It also has occasional contacts with other Competent Authorities in other Member States (Table 6). In parallel, schemes regularly (i.e., more than two times per year) assist the CAA in identifying systemic issues and sectoral problems through data returns and ad hoc data requests to identify any patterns of non-compliance. In particular, certified ADR entities must include in their annual reports information on any problems happening frequently and must make recommendations on how to solve them. Certified ADR entities therefore contribute to the work of the CAA acting as consumer protection agency. As the CAA has further highlighted, “the CAA welcomes this feedback as an important source of information for informing the prioritisation of its consumer protection work” (CAA 2017b). Interestingly, the CAA does not only request information from CAA-certified entities but may also–from time to time request information from other EU listed bodies “in order to inform its understanding of airlines compliance with the relevant consumer protection legislation in the aviation sector” (CAA 2016c).

##### Ongoing Developments in the Civil Aviation ADR Sector

The ADR aviation market in the UK is currently dominated by two players. The CAA has taken the view that, to facilitate signing-up to ADR schemes where signing-up to ADR schemes is voluntary, a choice of several ADR providers is necessary (Table 7). As a CAA’s Official stressed in the questionnaire:

“Our view has been that where ADR is voluntary, it is necessary to provide some choice for traders as regards cultural fit, IT systems, costs, etc. As long as high standards are maintained by focussed oversight by the Competent Authority this works well. It produces innovation for the market as a whole. We have examples of this such as different IT systems, feedbacks to traders etc. e.g. one of our ADR schemes has a better idea for gaining consumer feedback – rather than surveying consumers at the end of the complaints process (their comments tend to be closely related to whether they were successful). One ADR provider asks consumers question at different stages during the complaint process. We are planning to require this of the other ADR provider. This is a classic example of competitive forces freeing up creativity and then we can, as the regulator, push out further to the market to improve consumer outcomes. More than one provider gives a more robust framework potentially in case of company failure or traders pulling of schemes.^33^ It also provides the Competent Authority with more power to ensure effective outcomes etc rather than a single monopoly provider. If we had a single provider we would find it harder to rectify any issues because we would be so reliant upon them.”

CAA’s position is currently under review along with some other policy issues related to ADR, in particular the question of mandatory ADR.

#### ADR in Non-regulated Sectors

##### Background

The Secretary of State is the Competent Authority for ADR schemes operating in all non-regulated sectors. It has delegated its responsibilities in its role as Competent Authority to the Chartered Trading Standards Institute (CTSI), which also acts as single point of contact for the European Commission, and is in charge of coordinating the Competent Authorities group in the UK. BEIS’s call for evidence in 2011 indeed identified CTSI as one of the well-placed bodies for fulfilling this role as CTSI was already in charge of a similar certification duties for Consumer Codes Approval Schemes (CCAS). In spring 2018, CTSI had approved more than 30 schemes from various nature operating in a wide range of economic sectors, including package holidays and tourism, vehicle hire and lease, construction and maintenance, furniture, sports, transports etc. Noteworthy, CTSI has also approved a local authority (Kent County Council) as a certified ADR entity.^34^

##### Certification Process and Impacts on ADR Providers

CTSI provides guidance for applicants on its website. The approval process is informal, and the “audit process is designed to be dynamic and for the auditor to support the ADR towards approval” (CTSI 2016a). In practice, CTSI appoints an auditor to review the application and to help the applicant navigate certification requirements. Applications are reviewed based on the information transmitted by the applicant, onsite visits, and also sometimes feedbacks received from consumers or consumer organisations (Table 2). CTSI does not impose additional quality or performance criteria on schemes seeking certification (Table 4). The auditor answers any concerns and questions the applicant may have and clarifies the changes that are needed to the applicant’s structure and procedure. The auditor also decides, in consultation with the applicant, on a reasonable timeframe to implement the identified changes. An audit check is performed to ensure that the changes have been implemented correctly. If additional information is needed, the auditor can organise meetings with the applicant or request access to information at the applicants’ offices. Already in July 2016, CTSI reported the following impacts on ADR sectors (CTSI 2016b): ADR schemes had made significant improvements to their processes in order to comply with the new legal obligations, most applicants had for instance reduced their fees to consumers and a majority of them were now free to consumers. Approved bodies had also begun to work in sectors where there were no previous sector specific ADR bodies.

BEIS initial policy required that the costs of establishing CTSI as a Competent Authority not be a cost to the government. As a consequence, CTSI charges applicants a fee for certification and audits. The fee is structured on a cost recovery basis, and fees’ amounts must be communicated to the Secretary of State annually. In a letter sent to the Secretary of State in July 2016, CTSI explained its initial methodology for calculating fees (CTSI 2016a). The arrangement was initially composed of two parts: the first part covers the actual auditing of the applicant and applied a daily rate set at £750 (approximately €850). The amount due for this first part therefore depends on the size and complexity of the applicant. The second part was a periodic charge of £2000 per approval (approximately €2270). It was used to fund CTSI’s other activities relating to ADR, which included coordinating the Competent Authorities group, and acting as single contact point for the European Commission. In total, the average costs of individual audits for traders reached £4140 (approximately €4700) in 2016. However, BEIS highlighted some issues in the way fees had been structured. In particular, it considered that the £2000 periodic fees had “been used to fund certain functions that, while legitimate in CTSI’s role, should not have been funded by ADR providers under the terms of the ADR Regulations” (BEIS 2016). Consequently, BEIS decided to refund £2000 to all ADR providers. In parallel, CTSI was also concerned that the amounts of fees could deter ADR schemes from seeking certification. In particular, there was a risk that approved ADR schemes pass on the cost of audit in their charge to traders, which would ultimately discourage traders from using ADR schemes. As a consequence, BEIS ultimately agreed to provide some public funding to support CTSI in its role as Competent Authority (BEIS 2016). The objective was to encourage ADR bodies to become certified, and to lower the costs for traders taking part in ADR schemes. Taking these changes into account, the average approval costs now vary between £2500 and £3000 (approximately between €2840 and €3400) for traders. There are generally no further fees, unless non-compliance issues arise.

##### Challenges

Several difficulties were reporting as regards ADR certification (Table 3). Back in 2016, CTSI already highlighted that evaluating fees for consumers, and reviewing the qualification of ADR officials remain two key problems when reviewing certification applications. As it further explained, “experience has shown that these two issues are the issues where applicants ultimately refuse to meet the requirements of the legislation and pull out of the audit process” (CTSI 2016a). CTSI also reported that “the vast majority of approvals have been done without controversy and minimum impact. Feedbacks on these has been positive and most are now beginning to see the benefits of approval. This is however a first step and to date several business models have been tried and we are sure to see a rationalisation and possibly a simplification of the environment as new learning is incorporated” (CTSI 2016a).

##### Monitoring

The CTSI performs monitoring checks once a year always in collaboration with the scheme. It may also conduct a test case that is not expected (Table 5). Complaints from consumers and other stakeholders concerning an approved ADR body may also generate additional checks. As CTSI highlighted in its policy document, “the approval process creates an ongoing relationship with the Competent Authority” (CTSI 2016b) and certified schemes must inform the Authority in writing of any changes in their organization or structure.

##### Relationship Between the Competent Authority and Stakeholders

The CTSI has no contact with judges or the judiciary, and no contacts with Competent Authorities from other Member States (Table 6). Certified ADR schemes only occasionally (i.e., one or two times a year) assist the Authority in identifying systemic or sectoral problems. CTSI has regular contacts with other Competent Authorities in the UK. This is because the CTSI has been assigned the role of coordinating the Competent Authorities group in the UK. As it further highlighted, its role is to “coordinat[e] the Competent Authority group to ensure appropriate consistency going forward (this work will be essential to the robust delivery of the system and a lack of such a mechanism would leave the entire system open to public criticism)” (CTSI 2016a). In practice, CTSI and representatives of other Competent Authorities meet regularly, and generally have around four meetings a year (two face-to-face meetings and two teleconference calls). These meetings are generally at CTSI’s instigation but CTSI may also call a meeting if another Competent Authority suggest it for a particular reason. CTSI also frequently meets with individual Competent Authority. The talks generally deal with Best Practices for approved ADR bodies.

For the sake of comparison with the situation in other countries also relying on the multiple Competent Authorities model, Italy has created a coordination board (*Tavolo di coordinamento e di indirizzo*),^35^ linked to the Ministry of Economic Development (*Ministero dello Sviluppo Economico)* and composed of representatives of all Competent Authorities. It serves as a forum for exchanges and discussions. It defines policy orientations and clarifies possible ambiguities within the law and inconsistencies between practices.

##### Ongoing Developments of ADR in Non-regulated Sectors

CTSI has taken the view that a competitive market with several ADR schemes competing is beneficial since it can drive up standards, foster innovation, and offer greater choice to the sector (Table 7). As CTSI’s Chief Executive already explained in July 2016, “there need to be many more bodies approved so that competition drives innovation of model and a downward pressure on cost. The current and foreseeable environment requires awareness raising activity and a source of answers to enquiries to ensure this growth in competition” (CTSI 2016a).

## Looking to the Future

The Consumer ADR Directive has had some significant effects on the functioning and structures of ADR schemes in the surveyed Member States. As an attempt to look to the future, one first important question regards the needs to go beyond existing quality requirements so as to ensure higher levels of consumer protection (3.1). In parallel, several key issues still need to be resolved. They deal with the perceptions of consumers and traders vis-à-vis ADR quality, structures of ADR landscapes, and the role that Competent Authorities may have to play to further organize, rationalize and simplify ADR landscapes, as well as funding problems (3.2). Tools enabling information and knowledge sharing are particularly essential to further enhance the intervention of Competent Authorities (3.3).

### Going Beyond Existing Quality Requirements?

#### Impact of the Quality Requirements on Schemes’ Functioning in France and the UK: Overview

Let us go back to the concerns presented previously so as to further understand whether they indeed materialized in the surveyed Member States and sectors. All in all, in those countries, it appears that ADR quality has going through significant–albeit uneven–developments. One concern regarded the possible lack of effectiveness of the quality requirements listed under the Directive due to their vagueness and generality. In the two Member States here surveyed, it seems that the Consumer ADR Directive has to some extent contributed to step up schemes’ quality. The vagueness of the quality requirements as listed in the Directive has been counterbalanced by the careful scrutiny operated by Competent Authorities when certifying schemes (e.g., CECMC in France), or by the fact that Authorities may have imposed additional quality criteria for their sectors (e.g., CAA in the UK). As a result of certification, schemes have increased their transparency and visibility, and review their internal procedures. Some have hired new staff to ensure that the 90-day period for dealing with complaints could be respected. They have revised their websites and reviewed the way information is disclosed to consumers. Some of them also went through deeper structural changes. Certification processes and monitoring have also triggered discussions between Competent Authorities and schemes, sometimes leading to frictions as some schemes were not willing to change their practices easily. These frictions appear symptomatic of a transitional period in which all stakeholders must progressively learn new rules and adapt their behaviour to a new policy framework.

Another concern regarded the behaviour of Competent Authorities when certifying and monitoring ADR schemes. The three Competent Authorities have aided and guided ADR schemes a lot during certification phases. They helped them comply with the new legal requirements, and their approach has been mainly collaborative and supportive. However, the tasks of Competent Authorities were often more demanding and time-consuming than initially expected. Some of the difficulties can be explained by domestic contexts. For example, in France, difficulties were linked to the necessity to certify ADR schemes in all economic sectors in a short period of time (and in the absence of a residual ADR entity). Another issue regarded the necessity to carefully review the functioning and design of in-house mediators. Competent Authorities have conducted (or are progressively conducting) audits and control checks to verify that approved ADR schemes continuously comply with the requirements. These controls are expected to multiply in the future. One persisting question though regards the ability and capacity of Competent Authorities to perform subsequent control checks when many ADR schemes have been certified. This question will particularly be acute in France since the CECMC and DGCCRF will have to monitor–with limited resources and human capacities–more than 80 schemes (Bernheim-Desvaux 2017).

One issue also regarded the structure of monitoring architectures, and more specifically the effectiveness of decentralised architectures as the one existing in the UK. Although it may still be too early to draw up clear conclusions here, it nonetheless seems that CTSI is actively acting for ensuring homogeneity and consistency between Competent Authorities’ practices. However, a question regards CTSI’s capacity to maintain such efforts in the long run due to its limited resources. Homogeneity between practices might therefore still largely depends on the good will of other Competent Authorities. One possible tool for ensuring homogeneity between Competent Authorities might be to give to the coordinating Authority the possibility to make binding recommendations so as to ensure and facilitate coordination when needed (Biard 2018).

#### The Effectiveness of Quality Requirements: Views from Competent Authorities

The questionnaire investigated Competent Authorities’ s views on the capacity of their respective certification processes to allow for effective control over the performance and quality of ADR schemes. It also inquired whether, in Competent Authorities’ views, the quality requirements set down in their respective legislations have managed to ensure high-quality services for consumers (Tables 8 and 9). CTSI agreed that certification has permitted an effective control over the performance and quality of schemes. It also took the view that the quality requirements can ensure high-quality services for consumers. This is because they are flexible and can therefore easily adapt to the diversity of providers. The CAA also agreed that certification has permitted an effective control over schemes but also nuanced its statement and highlighted that this is because of the way certification has been organised and structured in the civil aviation sector, and in particular the existence of additional quality criteria. On this point, the CAA added that “it should be open for competent authorities to build in further criteria as they see fit for their sector. We have assured good outcomes for consumers by creating specific criteria for our sector.” Finally, the CECMC somehow agreed that certification allows for effective control over ADR schemes. However, the CMCE also took the view that existing requirements may not be sufficient to ensure high-quality services for consumers because they tend to remain too vague and do not necessarily fully capture the particularities of ADR providers. They should be augmented with other quality criteria and Key Performance Indicators.

For the sake of comparison, it should be noted that other Competent Authorities have adopted or plan to adopt requirements that go beyond what is currently required in their national legislations. For example, the Gambling Commission in the UK has stressed that “very few approved providers in the gambling sector appear to have a complaints process designed to accept complaints about their own services. Nor is this required in the ADR regulations” (Gambling Commission 2017). The Gambling Commission is currently reviewing its certification framework and standards. It may impose additional quality criteria, including on those schemes that are already certified (Gambling Commission 2017). Moreover, other sectors have developed rules to support access to ADR for vulnerable consumers. For example, OFGEM requires ADR schemes to show that they are able to provide a wide range of translation services for consumers who do not speak English or that they have adopted “processes that allow for additional help in accessing the scheme to be given to consumers that need it” (OFGEM 2015).

There is still experimentations ongoing at national levels concerning the type of quality requirements and the methods that are necessary for improving the quality of ADR. Several Competent Authorities have felt the need to go beyond existing quality requirements to offer higher services for consumers. Such different domestic approaches might widen the gap between ADR providers in the EU. This observation calls for higher consistency between national Competent Authorities. As hereafter highlighted, this is an area where the EU might have a role to play in the future.

### Focus on Persisting Issues

#### Consumers and Traders’ Perceptions Vis-à-Vis ADR Quality

The actual quality of ADR schemes may still not be perceived by consumers and traders. From the point of view of consumers, the lack of perceived independence of certain schemes continues to be an issue. Many of them are still dissatisfied about the way their complaints have been processed. In the UK, a 2018 study commissioned by BEIS pointed out that 46% of consumers using ADR had problems including concerns over the time the process took, customer service or a perception that the process favoured the business (BEIS 2018a). Another study published in 2017 raised similar problems relating to impartiality and fairness: 60% of surveyed consumers thought that ombudsmen were biased against them, 8% thought ombudsmen were biased towards them, while only 31% said that the ombudsmen were neutral. For only one ombudsman surveyed did the majority consider that the ombudsman was fair. For all others, more than 50% of users said the decision was not fair (MoneySavingExpert 2017). In parallel, in its annual report, the CAA highlighted that “there have been a few instances where consumers have questioned the independence and impartiality of the ADR provider, in particular where the ADR ruled against the consumer” (CAA 2017b).

From the viewpoint of traders, several of them appear still concerned about the way schemes operate. For example, in the aviation sector in the UK, the airline Jet2 expressed its scepticism in a letter sent to the CAA in which the company explained its decision to not adhere to an ADR scheme. Jet2 considered that the success rate for consumers in claims handled by one of the two approved schemes was “extraordinarily high”, and questioned whether the certified schemes could really be trusted. As Jet2 argued:

“Jet2.com is not satisfied with the quality of the decisionmakers or of the ADR providers which the CAA has sanctioned. For instance, we have seen criticism of the ability of TRO/Aviation ADR to deal with the large number of complaints it administers across numerous sectors. We have seen concerns expressed about the manner in which TRO has attracted corporate clients, which undermines its claims to independence: an ADR provider should be neutral, and be seen to be neutral, in all respects. Jet2.com is also concerned by the independence and quality of some of the adjudicators, who may have no judicial experience (and may not even be lawyers); may not be used to applying English, EU and/or international legislation, directives, regulations and conventions; may not be used to getting to grips with complex operational, technical and other factual issues; and may not be used to weighing up arguments and evidence or applying the correct evidential burden” (Jet2/CAA 2018, par 11).

One of the key objectives of certification was to act as a “trust-mark” incentivizing consumers and traders to use ADR schemes. If certification has been a first step for enhancing consumers’ and traders’ confidence, evidence tends to show that this is however still not sufficient. Additional measures should be taken to enhance transparency of ADR bodies. In this context, the CAA has for instance highlighted that it “intends to consider whether to enable the publication of data on the numbers of complaints received for each airline and the rate at which decisions are made in favour of the consumer”. It will also look at “whether there is any additional information which could be published about their independence and impartiality of the CAA approved ADR providers” (CAA 2017b). Similar evolutions are currently discussed by other ADR entities in other Member States. In practice, the publication of complaint data per trader may be a sensitive issue as ADR entities are also bound to respect confidentiality and privacy at all times during ADR procedures. Some political support and, where necessary, changes in legislations may be necessary for enhancing the disclosure of information while respecting confidentiality obligations. In parallel, another key question regards the way information about ADR schemes should be conveyed and communicated to consumers and traders. Behavioural research has indeed shown that simply increasing the level of available information might ultimately be counter-productive, as consumers may suffer from a so-called “information overload” problem (Ariely 2000; Scammon 1977). Enhancing transparency and trust may thus require reviewing the type and nature of the information communicated but also–and importantly–*how* this information is disclosed to consumers and traders.

#### Structures of ADR in France and the UK: A Search for Effective Architectures

##### Structures of ADR Landscapes in France and the UK: Suggested Typology

As exemplified by France and the UK, Member States are today navigating between different models. They are still searching for effective architectures likely to maximize the potential of ADR and supporting high-quality services for consumers and traders. Situations vary a lot not only between Member States but also between sectors within a same Member State. From the insights collected for France and the UK, three main types of ADR landscapes can be identified and described in economic terms (Table 1). They are, namely, competitive ADR markets (*Model 1*), oligopolistic models (*Model 2*) and monopolies (*Model 3*).

Schemes operating under Model 1 (competitive markets) compete to attract complaints. This is for instance the case in the banking sector in France and in the gambling, housing and non-regulated sectors in the UK. Oligopolistic markets are organized around a limited number of players. This is for instance the case in the civil aviation sector in the UK (two certified bodies to date) and in the energy sector in France (three certified bodies). Sometimes, some forms of cooperation have emerged between these players (e.g., collaboration agreements between ADR schemes in the French energy sector). Finally, monopolies are sectors dominated by one entity (often a public ombudsman or a sectoral entity), acting as a quasi-regulator. This is for instance the situation of the Financial Ombudsman Services in the UK.

Several Member States are currently searching for effective models likely to support high-quality ADR for consumers and traders. An effective ADR architecture should be able to offer high-quality services for consumers and traders at reasonable costs, be visible and easily understandable for all parties, maximize adherence from traders, facilitate effective monitoring by Competent Authority(ies) and fully exploit the potential of ADR for policymaking purposes (e.g., through exchanges between ADR schemes and regulators when systematic problems are unveiled). As Christopher Hodges has noted, “if CDR [consumer dispute resolution] schemes have particular design features, they can clearly support economic growth by performing three key features: (1) advice to consumers, (2) dispute resolution, (3) feeding back aggregated data on market trading conditions” (Hodges 2014, pp. 596–597).

The search for effective ADR structures is visible in the UK. As previously highlighted, some Competent Authorities (e.g., CTSI) have taken the view that competitive ADR markets (i.e., Model 1) is preferable because it can contribute to driving up quality standards, fostering innovation and can offer greater choice to sectors. Others (e.g., the Gambling Commission) initially supported competitive ADR markets, but have changed (or are in the process of changing) their views so as to support markets with limited competition (i.e., shift to Model 2). As the Gambling Commission has highlighted:

“We expected the relatively high number of approved ADR providers to provide consumers with more choice about which to use to deal with their complaint. Competition between providers could also have driven up standards of ADR provision. In practice, the anticipated competition has not materialised. Figures from the ADR providers’ first annual reports show that the bulk of disputes have gone to only two providers, and some providers dealt with no disputes over the year. In addition, because many providers have elected to specialise in one type of gambling, they do not compete with each other (…)” (Gambling Commission 2017).

In parallel, some oligopolistic markets (Model 2) are considering a shift towards monopolies (Model 3) in which only one ADR scheme operates, with no competition. This is the evolution discussed, for example, in the housing sector where four entities have been certified but each of them covers some aspects of buying and renting, but not all of them. Between February and April 2018, the Ministry of housing, communities and local government launched a consultation seeking views on “whether a single ombudsman service is needed to simplify access to redress across housing, and if so, what form that should take and what its remit should be” (Ministry of Housing, Communities and Local Government 2018). As the Community Secretary stressed, “this could help drive up standards across the whole industry and increase protections for consumers”. Policy discussions in the UK are now ongoing and will carefully contemplate the needs to introduce higher rationalisation and coherence within ADR sectors so as to avoid confusion and enhance consumers’ visibility.^36^ A 2018 Consumer Green Paper entitled “Modernizing Consumer Markets” also aimed to further clarify the matter. It pointed out:

“Some alternative dispute resolution providers and consumer representatives have questioned whether having more than one provider in a given sector is beneficial for consumers, suggesting that it could affect the quality of service provided as well as causing confusion to consumers. The government allowed for more than one provider in order to stimulate innovation and good value, giving businesses a choice of different types of dispute resolution at a range of costs. However, we would welcome your views on whether giving businesses a choice of provider is also beneficial to consumers” (BEIS 2018b).

Possible shifts from Model 1 to Model 2 (and in some cases, from Model 2 to Model 3) can therefore be expected in the UK. In other Member States, similar changes will depend on domestic ADR landscapes and political support. As discussed earlier, France’s initial plan was to organize its ADR landscape around key sectoral and public schemes. However, the plan turned out to be complicated because of the resistance of in-house mediators. It might revive in the future if the need for further rationalising the French ADR landscape becomes more pressing and is higher on the political agenda.

The benefits of having one single ADR entity per sector appear manifold (Hodges 2014, 2016, 2017). Among other things, sectoral schemes may better develop their market knowledge. They are familiar with businesses’ practices and with the problems faced by consumers. They know and can compare all practices of traders operating in the market. They have therefore a stronger authority and may better influence behaviour. Having one sectoral ADR scheme also facilitates the exchange of information and data with regulators. They may also be clear go-to points for consumers, thus reducing the risks of confusion. From the point of view of Competent Authorities, having one sectoral entity can contribute to narrow down and facilitate monitoring. This being said, interacting with only one single ADR scheme might also create a risk of capture of the Competent Authority by the scheme. News about FOS’s (Financial Ombudsman Service) activities in the UK–whose quality was seriously challenged by an investigation programme broadcasted on television in March 2018^37^–tend to question whether the controls operated by the Financial Conduct Authority acting as Competent Authority were in practice sufficient to ensure high-quality services.

##### Roles of Competent Authorities for Rationalizing and Simplifying ADR Markets

One important question is how higher rationalization and simplification within ADR landscapes should be carried out, in particular whether this should be left to market forces (meaning that ADR sectors would be able to structure themselves progressively around one or two key players, without external intervention), imposed by law, or influenced by Competent Authorities. It is interesting to note here that the preliminary discussions that took place in 2012 about the Consumer ADR Directive initially stressed that “it should be clear that authorities have no discretionary power to refuse inclusion in the list as long as the assessment shows that the ADR scheme respects the provisions in Chapter II” (EU Parliament 2012). The questionnaire thus aimed to understand whether, after several years of experience, Competent Authorities would actually appreciate to be given such discretionary powers allowing them to refuse certification when this appears necessary for structuring and simplifying ADR markets.

The questionnaire first investigated whether Competent Authorities had already refused certification to schemes that formally complied with the quality requirements, and whether they consider that they should be given discretionary powers to refuse certification (and if so, on which grounds or in which circumstances). Responses are presented in Tables 10 and 11. CTSI has, until now, never refused certification to schemes that complied with the quality requirements. It took the view that competent authorities should be given discretion to refuse certification when this is necessary for maintaining consumers’ visibility in a given sector or to facilitate monitoring. CAA has also not formally refused certification. However, some applicants withdrew on their own motion when they realised the extent of the additional requirements that were requested by the Authority. The CAA also considered that authorities should be given discretion to refuse certification, and further explained that “if competent have additional requirements which are published, then they can refuse on those grounds”. For the sake of comparison, it is interesting to note that the Gambling Commission in the UK is contemplating similar changes. It has notably highlighted:

“In 2017–18 we will look at reducing or limiting the number of approved ADR providers. We will revisit the requirements of the ADR regulations and consider how approval of an ADR provider might be supplemented with a framework of advice and measures to ensure a better experience for the consumer. This will include considering both additional service standards, a review of governance arrangements and independence, and requirements for decision making that make the role of an ADR provider much clearer. We will then review the approved ADR providers against the new framework. If providers fail to meet the increased standards, we will work with them to consider whether they wish to make changes to their procedures, or lose their approved status” (Gambling Commission 2017, p. 20).

The CECMC has never refused certification to schemes complying with the quality requirements. It also took the view that all ADR schemes complying with the quality requirements should be given equal access to certification. This response might be better explained by the peculiar relationship that the CECMC has with DGCCRF. One of the core functions of DGCCRF is indeed to guarantee market access and free competition between all economic actors. Giving the Authority some discretionary powers to restrict access to some schemes would thus go against the core role of DGCCRF.

#### Funding and ADR Quality

Finally, the issue of funding for ADR schemes is closely connected to the quality of ADR. Without sufficient resources, schemes will not meet expectations, and will not be able to provide high-quality services within the 90-day period. Staff is also less likely to possess the right and updated expertise to resolve complaints. In parallel, schemes’ funding arrangements can significantly alter the perceptions of consumers about the quality of services. For example, in 2017, the Gambling Commission reported that “some have voiced concern that an ADR provider cannot be independent if chosen and paid for by an operator, and we can see how this perception might arise” (Gambling Commission 2017). Measures are needed for stepping up schemes’ capacity while preserving their financial autonomy, in particular vis-à-vis traders. In Summer 2018, the EU Commission published several calls for grant aimed at assisting the development of ADR and ODR entities. One category of grants deals with capacity building and aims at assisting ADR entities wishing to develop their IT tools, training, promotion and also their awareness-raising, networking and mutual learning activities (EU Commission 2018a). A second category deals with grants for ADR entities, online traders and online marketplaces wishing to upgrade their software so as to facilitate work with the ODR platform (EU Commission 2018b).

### Tools for Enhancing Competent Authorities’ Intervention: Sharing Knowledge and Information

CECMC, CTSI and CAA were asked about possible tools likely to facilitate and enhance their intervention. Both CTSI and CAA highlighted the relevance of having a network of Competent Authorities at the EU level. Both CTSI and CECMC stressed the added value of Best Practices at the EU level. The CAA also stressed the importance of having a joined-up approach to sectoral legislation and ADR in the civil aviation sector^38^ (Table 12).

Some of these ideas have already been taken up by the EU Commission. For example, the first meeting gathering all EU Competent Authorities and the Commission is expected to take place for the first time at the end of 2018. In June 2018, the Commission also organised the first “ADR Assembly”, which brought together representatives of ADR entities, national Competent Authorities, European consumer centres, consumer associations, businesses representatives, ODR contact points and other stakeholders. One workshop was precisely dedicated to exploring and discussing challenges relating to ADR quality in the EU. Furthermore, in 2019, the European Commission is expected to publish a report on the implementation of the Consumer ADR Directive in the Member States. This report will evaluate the impact of the Directive and persisting problems. Finally, the EU Commission has also been encouraging discussions between stakeholders through the creation of various networks where ADR bodies and other stakeholders can discuss and exchange. In November 2017, TRAVEL-NET (the network of ADR schemes for transports, travel and tourism) was created. The launch event was hosted in Berlin at the Commission’s office and was attended by representatives of transport companies, consumers association and national authorities. Other networks like the National Energy Ombudsmen Network (NEON),^39^ and the Financial Dispute Resolution Network (FIN-NET)^40^ should also be noted. These networking and collaboration initiatives, supported at the EU level, are essential for sharing experience, enhancing coherence between Member States and ultimately stepping up consumer ADR quality throughout the EU.

## Policy Recommendations and Future Research

Based on the observations made throughout this paper, it is possible to make several policy recommendations to further strengthen the quality of ADR and the work of Competent Authorities. Although these recommendations are primarily relevant for France and the UK, they might also have broader implications.

1. Further developing and tailoring quality requirements. The quality requirements set down in the Consumer ADR Directive were a starting point. Active enforcement and monitoring by Competent Authorities have triggered some changes in the design and procedures of certified schemes. However, existing quality requirements are not enough. Competent Authorities should build in additional quality criteria tailored to the contexts and environments in which schemes operate. When doing so, they should consider the variety of consumers’ and traders’ profiles, and in particular the situation of vulnerable consumers.
2. Increasing resources of Competent Authorities*.* The effectiveness of the quality requirements depends on the active behaviour of Competent Authorities and their ability to supervise ADR markets carefully. Particular attention should be given to the resources (both financial and human) that Competent Authorities need to fulfil these tasks. Authorities with too little resources may not be able to live up expectations and may fail to monitor ADR.
3. Further structuring and rationalizing national ADR landscapes. Questions relating to architecture are closely connected to the question of quality. Calls for further rationalization of ADR sectors in the UK are not new (see, for example, Gill et al. 2017). They are currently discussed at policy level. A single sectoral scheme is likely to act as a clear go-to-point for consumers and traders and can help maximize the added value of ADR for consumer protection and policymaking. Competent Authorities may also have a role to play for simplifying ADR sectors. For example, imposing additional quality requirements or regularly updating requirements may result in a decrease in the number of certified schemes. Ultimately, this will ensure that only schemes offering the highest quality of services for consumers and traders continue to operate.
4. Perceptions of consumers and traders about ADR quality importantly matter. Paraphrasing the old saying, quality of schemes should not only exist but should also be seen to exist. The ADR Directive has increased and uniformized the type of information now disclosed by schemes. The objective was to increase trust and confidence among consumers and traders. However, this information has not always been sufficient to convince consumers and traders. Particular attention should be given to the type of information that really matter for consumers and traders, and–importantly–on how this information should be conveyed.
5. Sharing information and networking. With more than 45 Competent Authorities currently operating across the EU, practices of Competent Authorities vary a lot. Yet all of them may face similar issues during certification and monitoring phases. Exchanges of knowledge and Best Practices can assist Competent Authorities in their daily work. The EU Commission may have a key role to play here to ensure that Competent Authorities can learn from each other.

Building up high-quality ADR is still a work-in-progress today. As highlighted earlier, investigating the work of Competent Authorities is like shooting at a moving target: Competent Authorities are progressively accumulating experience, and asserting their roles in national ADR landscapes and vis-à-vis all stakeholders. Future research should carefully consider and compare the different approaches and actions taken by Competent Authorities for further rationalising ADR sectors and enhancing quality requirements. More generally, future research should also consider the roles that Competent Authorities might have to play for bridging formal and informal justice, which is a topic currently discussed by scholars and practitioners in the EU (ELI-ENCJ 2018, see also Biard 2018 for a suggestion). Ultimately, experiences from Competent Authorities in Member States represent key opportunities for cross-fertilisation. All together, these insights can contribute to enhance ADR quality in the EU.

## Appendix

**Table 1:** Suggested typology of ADR landscapes in France and the UK

Model 1: competitive markets (several players—high competition)	Model 2: oligopoly (limited number of players—limited competition)	Model 3: monopoly (1 player—no competition)
France: banking (+ 25 ADR schemes)	England: civil aviation (2), telecom (2)	England: financial services (1)
England: housing (4), gambling (11), other non-regulated sectors	France: energy (3)	

**Table 2:** Information used by competent authorities when reviewing certification applications

	Documentation submitted by schemes?	Hearings?	Onsite visits?	Feedbacks from consumers and/or consumer organizations?	Other sources of information?
CECMC	Always	Always	Always	(Not indicated)	(Not indicated)
CAA	Always	Sometimes	Sometimes	Always	Phone discussions
CTSI	Always	(Not indicated)	Always	Sometimes	(Not indicated)

*CECMC* Commission d’Évaluation et de Contrôle de la Médiation de la Consommation, *CAA* Civil Aviation Authority, *CTSI* Chartered Trading Standards Institute

**Table 3:** Degree of difficulty faced by competent authorities when reviewing quality requirements (e.g., because their review required in-depth discussions and/or additional information from the applicant)

	Reviewing the independence and impartiality of applicants	Reviewing the expertise of the applicant	Reviewing the transparency of the applicant	Reviewing the fairness of the applicant	Reviewing the accessibility of the applicant	Reviewing costs for consumers	Other issues/observations
CECMC	Often difficult	Sometimes difficult	Sometimes difficult	Often difficult	Sometimes difficult	Never difficult^a^	–^b^
CAA	Never difficult	Never difficult	Never difficult	Never difficult	Never difficult	Never difficult	–^c, d^
CTSI	Sometimes difficult	Never difficult	Sometimes difficult	Sometimes difficult	Never difficult	Often difficult	

*CECMC* Commission d’Évaluation et de Contrôle de la Médiation de la Consommation, *CAA* Civil Aviation Authority, *CTSI* Chartered Trading Standards Institute

^a^ADR must be free of charge for consumers in France

^b^Respondent indicated that the economic model adopted by the scheme is essential as the services offered should not be too expensive for traders, but still sufficiently high to ensure the viability of the scheme. On fairness, the respondent added that the authority cannot control the fairness of the decision that is taken by the scheme for confidentiality reasons.

^c^Respondent for CAA further indicated: “it wasn’t easy and took a lot of time because we have very specific rule–for example, exact wording with needs to go into scheme rules. [We] had to assist applicants a lot. However, your question related to assessing the Directive criteria which is fairly straightforward”

^d^Respondent for CAA further indicated: “on transparency, (.) any requirements on consumers to keep their ADR discussions/outcome confidential should be prohibited under the directive. We have not permitted it as part of any scheme rules as we want it all to be transparent rather than being seen as secretive”

**Table 4:** Does the Competent Authority impose additional performance or quality requirements on certified providers?

	Yes/no	Observations
CECMC	Yes/no	Respondent clarified that no additional quality requirements are imposed at the time of certification, but they may be afterwards, during the monitoring phase.
CAA	Yes	See full policy here CAP1324 publicapps.caa.co.uk/modalapplication.aspx?appid=11&mode=detail&id=6819
CTSI	No	

*CECMC* Commission d’Évaluation et de Contrôle de la Médiation de la Consommation, *CAA* Civil Aviation Authority, *CTSI* Chartered Trading Standards Institute

**Table 5:** Monitoring performed by Competent Authorities

	Periodicity	By surprise/in collaboration with schemes	Other checks?
CECMC	Once a year	Always in collaboration with the scheme	Complaints received may trigger additional checks
CAA	Once a year formal “continuation of approval”	Both	Ongoing continuous oversight, including quarterly meetings, frequent data returns
CTSI	Once a year	always in collaboration (CTSI may do a test case that is not expected)	Regular contacts will all approved ADR bodies. Complaints received from consumers/others regarding an approved body would generate further checks

*CECMC* Commission d’Évaluation et de Contrôle de la Médiation de la Consommation, *CAA* Civil Aviation Authority, *CTSI* Chartered Trading Standards Institute

**Table 6:** Contacts and exchanges between competent authorities and stakeholders

	With judges/judiciary?	With other national competent authorities?	With other EU competent authorities?	With sectoral regulators?	With certified ADR schemes (highlighting sectoral or systemic problems)?
CECMC	Yes, occasionally (1 or 2 times a year)	n/a^a^	Yes, occasionally (1 or 2 times a year)	Yes, regularly (more than 2 times a year)	Yes, regularly (more than 2 times a year)
CAA	No	Yes, occasionally (1 or 2 times a year)	Yes, occasionally (1 or 2 times a year)	Yes, regularly (more than 2 times a year)	Yes, regularly (more than 2 times a year)
CTSI	No	Yes, regularly (more than 2 times a year)	No	Yes, occasionally (1 or 2 times a year)	Yes, occasionally (1 or 2 times a year)

*CECMC* Commission d’Évaluation et de Contrôle de la Médiation de la Consommation, *CAA* Civil Aviation Authority, *CTSI* Chartered Trading Standards Institute

^a^There is only one Competent Authority in France

**Table 7:** Competent Authorities’ views on the benefit/inconvenient of having multiple ADR schemes per sector

	Preference for multiple or limited number of schemes per sector	Reasons
CECMC	Nuanced (depends on sectors)	Multiple schemes may foster competition but added value of sectoral schemes
CAA	Multiple (because signing up to ADR schemes is currently voluntary in the aviation sector)	Foster competition drive-up quality standards avoid dealing with a single monopoly provider^a^
CTSI	Multiple	Drive-up quality standards foster competition offer greater choices to the sector

*CECMC* Commission d’Évaluation et de Contrôle de la Médiation de la Consommation, *CAA* Civil Aviation Authority, *CTSI* Chartered Trading Standards Institute

^a^Respondent added: “our view has been that where ADR is voluntary, it is necessary to provide some choice for traders as regards cultural fit, IT systems, costs, etc. As long as high standards are maintained by focussed oversight by the Competent Authority this works well. It produces innovation for the market as a whole. We have examples of this such as different IT systems, feedbacks to traders etc., e.g., one of our ADR schemes has a better idea for gaining consumer feedback–rather than surveying consumers at the end of the complaints process (their comments tend to be closely related to whether they were successful). One ADR provider asks consumers question at different stages during the complaint process. We are planning to require this of the other ADR provider. This is a classic example of competitive forces freeing up creativity and then we can, as the regulator, push out further to the market to improve consumer outcomes. More than one provider gives a more robust framework potentially in case of company failure or traders pulling of schemes. It also provides the Competent Authority with more power to ensure effective outcomes etc. rather than a single monopoly provider. If we had a single provider, we would find it harder to rectify any issues because we would be so reliant upon them”

**Table 8:** Effectiveness of quality requirements (1)Does the certification scheme allow for an effective control over the performance and quality of Consumer ADR providers?

	Strongly disagree	Disagree	Somehow disagree	No opinion	Somehow agree	Agree	Strongly agree
CECMC					X		
CAA						X (because of the way it has been set up in the UK aviation sector)	
CTSI						X	

*CECMC* Commission d’Évaluation et de Contrôle de la Médiation de la Consommation, *CAA* Civil Aviation Authority, *CTSI* Chartered Trading Standards Institute

**Table 9:** Effectiveness of quality requirements (2)Do the quality requirements for ADR providers set out in your legislation manage to ensure high-quality services for consumers?

	Yes/no	Reason(s)
CECMC	No	They are too vague and do not fully capture the particularities of ADR providers. They should be augmented with other quality criteria and Key Performance Indicators.
CAA	Respondent replied: “It should be open for competent authorities to build in further criteria as they see fit for their sector. We have assured good outcomes for consumers by creating specific criteria for our sector—see guidance: CAP1324.”	
CTSI	Yes	They are flexible and adapt easily to the diversity of ADR providers.

**Table 10:** Has your Competent Authority already refused certification to schemes that formally complied with the quality requirements?

	Yes/no	Observations
CECMC	No	
CAA	No	Respondent added: “we have not formally refused but applicants have withdrawn when they have realised the extent of our requirements and the practical work involved in the sector in which we operate”.
CTSI	No	

*CECMC* Commission d’Évaluation et de Contrôle de la Médiation de la Consommation, *CAA* Civil Aviation Authority, *CTSI* Chartered Trading Standards Institute

**Table 11:** Should Competent Authorities be given discretionary powers to limit the number of schemes per sector?

	Yes/no	Reasons/observations
CECMC	No	All schemes complying with the requirements should be given equal access to certification
CAA	Yes	Respondent added: “if competent authorities have additional requirements which are published then they can refuse on those grounds. We have done so. Without having additional requirements there would have been little advantage to consumers to having introduced ADR to our market as it may not have improved outcomes for them”.
CTSI	Yes	For maintaining consumers’ visibility in the sector; for facilitating monitoring

*CECMC* Commission d’Évaluation et de Contrôle de la Médiation de la Consommation, *CAA* Civil Aviation Authority, *CTSI* Chartered Trading Standards Institute

**Table 12:** Tools for strengthening Competent Authorities’ intervention in the future

CECMC	Elaboration of best practices at the EU level
CAA	Network of Competent Authorities at the EU level; joined-approach combining sectoral legislations and ADR
CTSI	Elaboration of best practices at the EU level; network of Competent Authorities

*CECMC* Commission d’Évaluation et de Contrôle de la Médiation de la Consommation, *CAA* Civil Aviation Authority, *CTSI* Chartered Trading Standards Institute

## Footnotes

1 Directive 2013/11/EU of the European Parliament and of the Council of 21 May 2013 on alternative dispute resolution for consumer disputes and amending Regulation (EC) No. 2006/2004 and Directive 2009/22/EC.

2 Art. 2(3) of Directive 2013/11/EU.

3 Art. 26 of Directive 2013/11/EU.

4 Art. 5 to 11 of Directive 2012/11/EU.

5 Art. 18 of Directive 2013/11/EU.

6 Art. 18 to 20 of Directive 2013/11/EU.

7 Recital 26 of Directive 2013/11/EU.

8 As explained below, some Member States (e.g., Italy, UK) have appointed several Competent Authorities.

9 The questionnaire was sent to Competent Authorities in the following countries: Austria, Belgium, Czech Republic, Denmark, Estonia, Finland, France, Germany, Hungary, Ireland, Italy, Latvia, Luxembourg, Malta, Poland, Romania and the UK.

10 Belgium and Luxembourg also took part in the study. Data collection and analysis of responses are still ongoing at this stage.

11 Despite several attempts, the other UK Competent Authorities did not reply to the survey.

12 Art. L611-1 *et seq* of French consumer Code (*Code de la consommation*).

13 Art. L613-1 of consumer Code.

14 Art. L612-1 of consumer Code.

15 Art. L616-1 and L612-1 of consumer Code.

16 www.economie.gouv.fr/mediation-conso/liste-des-mediateurs-consommation.

17 Art. L613-2 of consumer Code.

18 Art. L615-1 *et seq* and R615-1 *et seq* of consumer Code. The CECMC has replaced the former *Commission de Médiation de la Consommation* (CMC) created in 2010, and the *Comité de la Médiation Bancaire* since these two entities were no longer compliant with the requirements of the Consumer ADR Directive (Constans 2014).

19 Art. R615-1 and R615-2 of consumer Code.

20 www.economie.gouv.fr/mediation-conso/commission/deposez-dossier-mediateur-consommation.

21 Keynote speech of the CECMC’s President delivered at the event “2 ans de médiation de la consommation en France et en Europe”, organised by Médiateur National de l’Energie, 9 January 2018 (text kindly sent by Ms. Nocquet).

22 Data kindly sent by CECMC (July 2018).

23 www.economie.gouv.fr/files/Modele_de_convention-type_CECMC.pdf.

24 The information was available on CECMC’s Website, and then removed once the requested changes were made by the scheme.

25 Compromis sur la transposition de la directive mediation, 30 March 2015, www.sosconso.blog.lemonde.fr/2015/03/30/compromis-sur-la-transposition-de-la-directive-mediation/(translation by the author).

26 Art. L612-1 of Consumer Code.

27 Art. L612-2 3° of Consumer Code.

28 Art. L122-1 of energy Code (*Code de l’énergie*).

29 The lead enforcement authority for the purposes of the Estate Agents Act.

30 www.ted.europa.eu/TED/notice/udl?uri=TED:NOTICE:394225-2014:TEXT:EN:HTML&src=0.

31 www.caa.co.uk/Our-work/About-us/Alternative-Dispute-Resolution/.

32 Ombudsman Services and Net Neutrals gave up their approval respectively in May 2016 and July 2017.

33 In November 2015, Ryanair signed up to the Ombudsman Services (OS), but OS decided to withdraw from the aviation market few months later (Ombudsman Services, “Ryanair: the first airline signed up Ombudsman Services”, 23 November 2015, www.ombudsman-services.org/news/news-item/2015/11/23/ryanair-the-first-airline-signed-up-to-ombudsman-services.

34 www.localgov.co.uk/Kent-first-approved-council-to-provide-Alternative-Dispute-Resolution-services/41426.

35 Art. 141 octies of Italian consumer Code (*Codice del Consumo*) www.sviluppoeconomico.gov.it/images/stories/documenti/Tavolo_coordinamento_indirizzo.pdf.

36 In March 2018, Financial Times reported a growing “dissatisfaction (…) about the ineffectiveness of the UK’s complex ombudsman system” (Financial Times, “Complaints put consumer watchdogs on watch”, 16 March 2018).

37 Channel 4, “Investigation at FOS finds staff with severe lack of training”, 12 March 2018, www.channel4.com/info/press/news/investigation-at-fos-finds-staff-with-severe-lack-of-training.

38 Respondent referred in particular to the relation between National Enforcement Bodies (NEBs) under EC Regulation 261/2004 (Regulation EC 261/2004 establishing common rules on compensation and assistance to passengers in the event of denied boarding and of cancellation or long delay of flights) and their interaction with ADRs.

39 www.neon-ombudsman.org/.

40 http://www.ec.europa.eu/info/business-economy-euro/banking-and-finance/consumer-finance-and-payments/consumer-financial-services/financial-dispute-resolution-network-fin-net_en.
